# Unveiling Anti-Diabetic Potential of Baicalin and Baicalein from Baikal Skullcap: LC–MS, In Silico, and In Vitro Studies

**DOI:** 10.3390/ijms25073654

**Published:** 2024-03-25

**Authors:** Wencheng Zhao, Huizi Cui, Kaifeng Liu, Xiaotang Yang, Shu Xing, Wannan Li

**Affiliations:** Edmond H. Fischer Signal Transduction Laboratory, Key Laboratory for Molecular Enzymology and Engineering of Ministry of Education, School of Life Sciences, Jilin University, Changchun 130012, China; wczhao16@mails.jlu.edu.cn (W.Z.); hzcui23@mails.jlu.edu.cn (H.C.); liukf1220@mails.jlu.edu.cn (K.L.); yangxt22@mails.jlu.edu.cn (X.Y.)

**Keywords:** network pharmacology, LC–MS, cellular assay, molecular docking, PTP1B, anti-diabetic mechanisms

## Abstract

Type 2 diabetes mellitus (T2DM) is marked by persistent hyperglycemia, insulin resistance, and pancreatic β-cell dysfunction, imposing substantial health burdens and elevating the risk of systemic complications and cardiovascular diseases. While the pathogenesis of diabetes remains elusive, a cyclical relationship between insulin resistance and inflammation is acknowledged, wherein inflammation exacerbates insulin resistance, perpetuating a deleterious cycle. Consequently, anti-inflammatory interventions offer a therapeutic avenue for T2DM management. In this study, a herb called Baikal skullcap, renowned for its repertoire of bioactive compounds with anti-inflammatory potential, is posited as a promising source for novel T2DM therapeutic strategies. Our study probed the anti-diabetic properties of compounds from Baikal skullcap via network pharmacology, molecular docking, and cellular assays, concentrating on their dual modulatory effects on diabetes through Protein Tyrosine Phosphatase 1B (PTP1B) enzyme inhibition and anti-inflammatory actions. We identified the major compounds in Baikal skullcap using liquid chromatography–mass spectrometry (LC–MS), highlighting six flavonoids, including the well-studied baicalein, as potent inhibitors of PTP1B. Furthermore, cellular experiments revealed that baicalin and baicalein exhibited enhanced anti-inflammatory responses compared to the active constituents of licorice, a known anti-inflammatory agent in TCM. Our findings confirmed that baicalin and baicalein mitigate diabetes via two distinct pathways: PTP1B inhibition and anti-inflammatory effects. Additionally, we have identified six flavonoid molecules with substantial potential for drug development, thereby augmenting the T2DM pharmacotherapeutic arsenal and promoting the integration of herb-derived treatments into modern pharmacology.

## 1. Introduction

The rapid increase in the prevalence of diabetes is a serious public health crisis, with approximately 500 million patients worldwide. It is estimated that, by 2045, the global diabetic population will astonishingly increase by 51% [1]. This rise is not merely a leading cause of death but also imposes a substantial economic impact on healthcare systems due to the costs associated with treatment and the multitude of ensuing complications [2]. Type 2 diabetes mellitus (T2DM) is a chronic metabolic ailment that derives from insulin resistance and compromised insulin secretion, perpetuating chronic hyperglycemia [3]. T2DM is often linked to multi-organ dysfunction and is significantly associated with cardiovascular maladies, particularly atherosclerosis [4]. Inflammation within adipose tissue and gut microbiota imbalances contribute to insulin resistance, while impaired pancreatic β-cells struggle to regulate blood sugar effectively [5,6]; thus, the pathophysiology of T2DM is deeply intertwined with complex metabolic disruptions and inflammatory processes, with inflammation playing a pivotal role [7]. Inflammation within the pancreatic islets, for instance, can severely impair β-cell function in T2DM sufferers [8]. The inflammatory pathways involved in diabetes are increasingly recognized as potential focal points for advancing disease prevention and management [9]. Protein Tyrosine Phosphatase 1B (PTP1B) is an enzyme of paramount importance. PTP1B regulates the glucose levels in the bloodstream by catalyzing the dephosphorylation of tyrosine residues in the insulin receptor signaling pathway, effectively halting insulin signaling [10]. This regulatory mechanism is crucial for maintaining glucose homeostasis and highlights the enzyme’s significance as a potential target for therapeutic intervention in diabetes management [11].

*Scutellaria baicalensis*, commonly referred to as Baikal skullcap, is a Chinese herb lauded for its extensive pharmacological attributes [12]. Traditionally celebrated for its ability to clear heat, dry dampness, purge fire, detoxify, and arrest bleeding, it is also revered for providing cardiovascular and hepatic support, as well as bolstering immune functions [13]. It contains bioactive constituents such as baicalin, baicalein, and chrysin, which amplify its therapeutic efficacy. The utilization of Baikal skullcap is widespread, serving as a complementary remedy for a variety of ailments including inflammation, hypertension, cancer, and viral infections [14,15], and it is particularly noteworthy for its beneficial effects on diabetes and inflammation-related conditions. Nevertheless, the precise mechanisms through which Baikal skullcap aids in diabetes management warrant further exploration. The effectiveness of herbs is often credited to their active ingredients, a concept that is gaining credence through contemporary pharmacological research [16]. The isolation of these active ingredients is vital for curtailing the possible adverse effects associated with the use of the entire herb. Natural derivatives like the antimalarial artemisinin [17] and the anticancer drug paclitaxel [18] exemplify the benefits of elucidating and refining active components to enhance their medicinal properties. The derivation of aspirin from salicylic acid via chemical modification is indicative of the potential benefits of such research endeavors [19].

**Figure 1:**
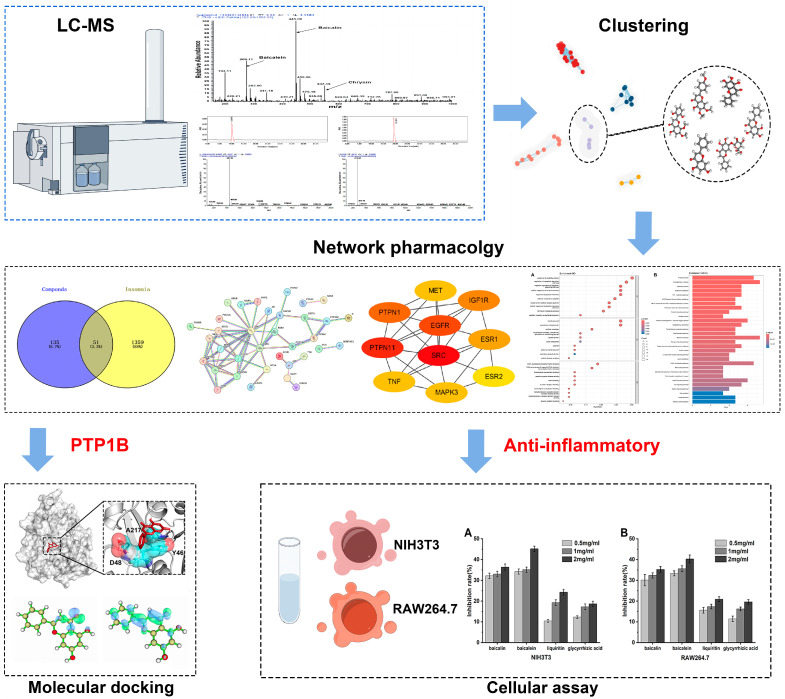
The workflow of our study.

In this study, we employed network pharmacology and cellular assays to probe the intricate interactions by which the primary bioactive constituents of Baikal skullcap might mitigate T2DM. We utilized LC–MS and the TCMSP database [20] to identify and screen for effective active small molecules in Baikal skullcap, upon which we conducted cluster analysis [21]. We have identified six flavonoid molecules that exhibit potential for pharmaceutical development due to their dual function in inhibiting the PTP1B enzyme and manifesting anti-inflammatory effects. Subsequently, molecular docking was employed to confirm the inhibitory effect on PTP1B, and cell experiments further validated the anti-inflammatory effects. We also compared the anti-inflammatory efficacy of Baikal skullcap with that of recognized anti-inflammatory drug glycyrrhiza [22]. The workflow is shown in Figure 1. Our research not only deepens our comprehension of T2DM management but also underscores the untapped potential of TCM in rectifying complex metabolic imbalances. 

## 2. Results

### 2.1. Identification and Screening of Effective Active Small Molecules in Baikal Skullcap

**Figure 2:**
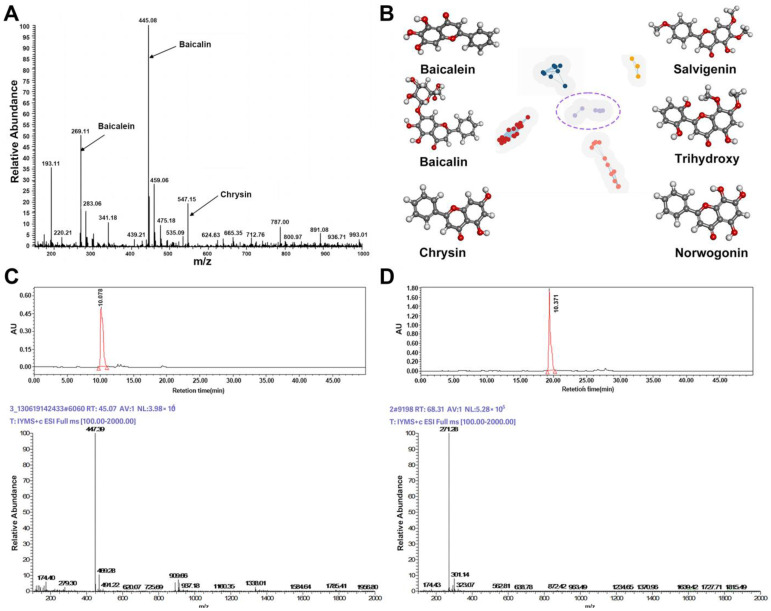
Identification and screening of effective active small molecules in Baikal skullcap. (**A**): Mass spectrometry detection of Baikal skullcap extracts. (**B**): Six active small molecules in Baikal skullcap from cluster analysis. (**C**): The liquid phase and mass spectrum of baicalein. (**D**): The liquid phase and mass spectrum of baicalin.

As shown in (Figure 2A), the major components identified in the Baikal skullcap extract include baicalein, baicalin, and chrysin, with baicalein and baicalin being the principal compounds. Mass spectrometric analysis, as depicted in Figure 2C,D, indicated that the main constituents of baicalein (molecular weight 270.24) and baicalin (molecular weight 446.35) were isolated from Baikal skullcap. The components and their purities in the purchased pharmaceuticals were characterized using LC–MS (Figure 2), illustrating the molecular weight and purity validation of the main components, baicalein and baicalin, in the acquired Baikal skullcap extracts, confirming compliance with the prescribed standards. We have identified liquiritin and glycyrrhizic acid in licorice using the same method (Figures S1 and S2). Subsequently, we integrated the molecular data of Baikal skullcap from the TCMSP database, and, based on the similarity of the molecular structures, conducted a cluster analysis to identify three additional components from *Scutellaria baicalensis* in the TCMSP database. In total, six components were identified for further analysis, resulting in the clustering (Figure 2B) of six molecules, whose information is shown in Table 1.

### 2.2. Identification of T2DM-Related Targets and Active Compounds in Baikal Skullcap

From an extensive review of the DisGeNET, GeneCards, and PharmGKB databases, we identified 1410 genes implicated in the pathogenesis of type 2 diabetes mellitus (T2DM). A subsequent analysis of the pharmacological targets associated with various bioactive compounds, including chrysin, salvigenin, and baicalein, among others, highlighted a subset of 51 to 59 genes that intersect with T2DM pathways, as delineated in Venn diagrams (Figure S3). Further exploration through protein–protein interaction (PPI) networks accentuated the interconnected nature of these targets (Figure S4).

### 2.3. Stratification of Key Targets in Baikal Skullcap’s Anti-T2DM Mechanisms

**Figure 3:**
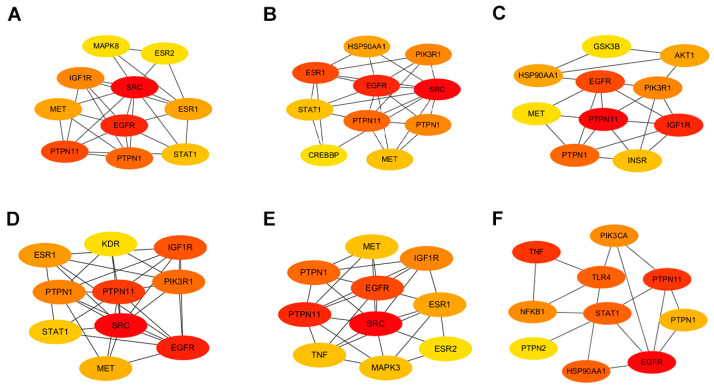
Protein interactions involved in Baikal skullcap treatment of T2DM. (**A**): The interaction of core targets of chrysin. (**B**): The interaction of core targets of salvigenin. (**C**): The interaction of core targets of 5,2′,6′-trihydroxy-7,8-dimethoxyflavone. (**D**): The interaction of core targets of norwogonin. (**E**): The interaction of core targets of baicalein. (**F**): The interaction of core targets of baicalin.

Employing the Maximal Clique Centrality (MCC) algorithm within the cytoHubba toolkit allowed us to stratify the pivotal nodes within the Baikal skullcap–T2DM interactome. The MCC scores, reflecting the connectivity strength, were visually represented with varying color intensities, with a deeper hue signifying a higher relevance to T2DM pathogenicity. Subsequently, we cataloged the top 10 targets for each active small molecule, unveiling key players such as the *PTPN1* gene encoding the PTP1B protein and the TNF target associated with inflammation (Figure 3).

### 2.4. Functional Enrichment of Baikal Skullcap’s Intersection with T2DM

We conducted Gene Ontology (GO) and Kyoto Encyclopedia of Genes and Genomes (KEGG) pathway enrichment analyses on the shared genes between Baikal skullcap and T2DM. The results, presented as bubble charts and histograms, unveiled a spectrum of biological processes, including the regulation of apoptotic pathways and responses to oxidative stress, alongside cellular component involvement such as membrane rafts and vesicle lumens (Figures S5–S10). The KEGG pathway analysis exposed several critical pathways, including those related to prostate cancer, endocrine resistance, and insulin signaling pathways, further elucidating the molecular interplay between Baikal skullcap constituents and T2DM (Figures S5–S10). Particularly within the KEGG pathways, all six active small molecules were enriched in one or multiple inflammation-related pathways, such as the IL-17 signaling pathway, TNF signaling pathway, MAPK signaling pathway, and PI3K-Akt signaling pathway. Notably, baicalin and baicalein have a greater number of targets associated with inflammatory pathways, leading us to speculate that there is a close connection between inflammation and diabetes.

Regulation of the apoptotic signaling pathway, response to oxidative stress, negative regulation of the apoptotic signaling pathway, and others were identified as potentially relevant biological processes related to the core targets of chrysin (Figure S5). The parts of enriched cell components included membrane raft, membrane microdomain, vesicle lumen, transferase complex, transferring phosphorus, secretory granule lumen, and cytoplasmic vesicle lumen. Additionally, the following molecular functions were identified by GO enrichment analysis: flavin adenine dinucleotide binding, steroid binding, neurotransmitter receptor activity, and others (Figure S5A). The enrichment pathways identified following KEGG analysis that contained the most core targets included prostate cancer, endocrine resistance, proteoglycans in cancer, adherens junction, insulin resistance, the IL-17 signaling pathway, the PI3K-Akt signaling pathway, and others (Figure S5B).

Response to peptide hormone, cellular response to peptide, positive regulation of protein localization, and others were identified as biological processes related to the core targets of salvigenin (Figure S6). The parts of cell components that were enriched included membrane raft, membrane microdomain, vesicle lumen, secretory granule lumen, and others. Additionally, the following molecular functions were identified by GO enrichment analysis: RNA polymerase II-specific DNA-binding transcription factor binding, transcription coregulator binding, serine hydrolase activity, and others (Figure S6A). The enrichment pathways identified following KEGG analysis that contained the most core targets included prostate cancer, fluid shear stress and atherosclerosis, prolactin signaling pathway, proteoglycans in cancer, PD-L1 expression and the PD-1 checkpoint pathway in cancer, the relaxin signaling pathway, the IL-17 signaling pathway, and more (Figure S6B).

Cellular response to chemical stress, response to peptide hormone, response to oxidative stress, negative regulation of apoptotic signaling pathway, and others were identified as biological processes related to the core targets of 5,2′,6′-trihydroxy-7,8-dimethoxyflavone (Figure S7). The parts of cell components that were enriched included vesicle lumen and others. Additionally, the following molecular functions were identified by GO enrichment analysis: hormone binding, heme binding, protein phosphatase binding, tetrapyrrole binding, and others (Figure S7A). The enrichment pathways identified following KEGG analysis that contained the most core targets included prostate cancer, the HIF-1 signaling pathway, insulin resistance, the IL-17 signaling pathway, the PI3K-Akt signaling pathway, the TNF signaling pathway, and more (Figure S7B). 

Cellular response to chemical stress, response to oxidative stress, regulation of apoptotic signaling pathway, response to peptide hormone, and others were identified as biological processes related to the core targets of norwogonin (Figure S8). The parts of cell components that were enriched included membrane raft, membrane microdomain, nuclear envelope, and others. Additionally, the following molecular functions were identified by GO enrichment analysis: hormone binding, transcription coregulator binding, protein tyrosine kinase activity, and others (Figure S8A). The enrichment pathways identified following KEGG analysis that contained the most core targets included proteoglycans in cancer, prostate cancer, endocrine resistance, the PI3K-Akt signaling pathway, and more (Figure S8B).

Response to oxidative stress and others were identified as biological processes related to the core targets of baicalein (Figure S9). The parts of cell components that were enriched included membrane raft, membrane microdomain, nuclear envelope, vesicle lumen, and others. Additionally, the following molecular functions were identified by GO enrichment analysis: DNA-binding transcription factor binding, RNA polymerase II-specific DNA-binding, transcription factor binding, transcription coregulator binding, and more (Figure S9A). The enrichment pathways identified following KEGG analysis that contained the most core targets included prostate cancer, proteoglycans in cancer, adherens junction, the PI3K-Akt signaling pathway, the MAPK signaling pathway, and more (Figure S9B).

Regulation of body fluid levels, response to mechanical stimulus, positive regulation of cytokine production, response to hypoxia, and others were identified as biological processes related to the core targets of baicalin (Figure S10). The parts of cell components that were enriched included membrane raft, membrane microdomain, vesicle lumen, and others. Additionally, the following molecular functions were identified by GO enrichment analysis: ubiquitin-like protein ligase binding, ubiquitin protein ligase binding, protein phosphatase binding, and more (Figure S10A). The enrichment pathways identified following KEGG analysis that contained the most core targets included lipid and atherosclerosis, the C-type lectin receptor signaling pathway, the IL-17 signaling pathway, Kaposi sarcoma-associated herpesvirus infection, Chagas disease, the HIF-1 signaling pathway, the TNF signaling pathway, and more (Figure S10B). After network construction and enrichment, we zeroed in on key PTP1B targets and anti-inflammation.

### 2.5. Quantum Chemical Analysis of Baikal Skullcap Constituents

**Figure 4:**
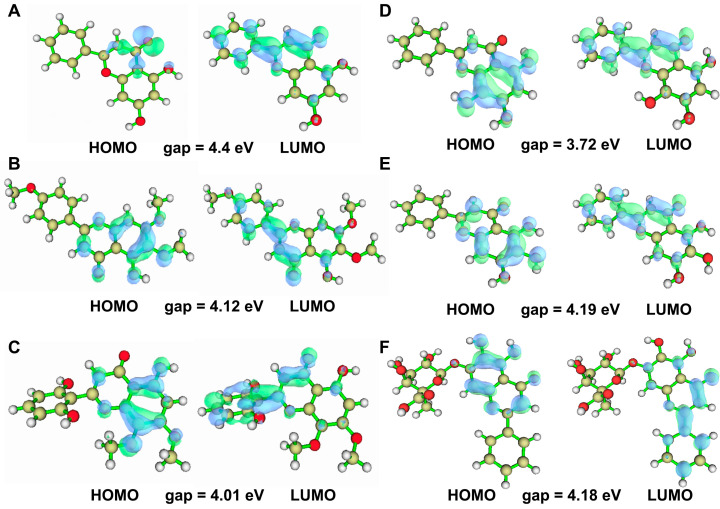
HOMO–LUMO orbital diagram of the six active ingredient molecules of Baikal skullcap. (**A**): The HOMO–LUMO orbital diagram of chrysin. (**B**): The HOMO–LUMO orbital diagram of salvigenin. (**C**): The HOMO–LUMO orbital diagram of 5,2′,6′-trihydroxy-7,8-dimethoxyflavone. (**D**): The HOMO–LUMO orbital diagram of norwogonin. (**E**): The HOMO–LUMO orbital diagram of baicalein. (**F**): The HOMO–LUMO orbital diagram of baicalin.

Quantum chemical calculations were performed using DFT/B3LYP at the 6–31G basis set level with Gaussian 09 to elucidate the electronic properties of Baikal skullcap constituents. HOMO (Highest Occupied Molecular Orbital) and LUMO (Lowest Unoccupied Molecular Orbital) are used in molecular orbital theory to describe the quantum mechanical states of electrons in a molecule. The HOMO and LUMO orbitals are almost directly related to nucleophilic and electrophilic attacks, respectively. Thus, visualizing these orbital characteristics, in addition to resolving the atomic contributions within the target pocket, serves as a valuable tool for exploring binding profiles. Therefore, the computation of HOMO and LUMO orbitals facilitates the strategic design of molecules. The HOMO–LUMO gap calculations revealed a range of values indicative of the electronic stability and reactivity potential of these compounds (Figure 4). The calculation results of the HOMO–LUMO gap (Figure 4) were (1) chrysin: HOMO–LUMO gap = −4.4 eV, (2) salvigenin: HOMO–LUMO gap = −4.12 eV, (3) 5,2′,6′-trihydroxy-7,8-dimethoxyflavone: HOMO–LUMO gap = −4.01 eV, (4) norwogonin: HOMO–LUMO gap = −3.72 eV, (5) baicalein: HOMO–LUMO gap = −4.19 eV, and (4) baicalin: HOMO–LUMO gap = −4.18. We evaluated the HOMO–LUMO gap, assessing the relative stability of six ligand compounds. Particularly, baicalein, baicalin, and salvigenin exhibit larger gaps, indicating comparatively higher stability.

### 2.6. Molecular Docking Studies

**Figure 5:**
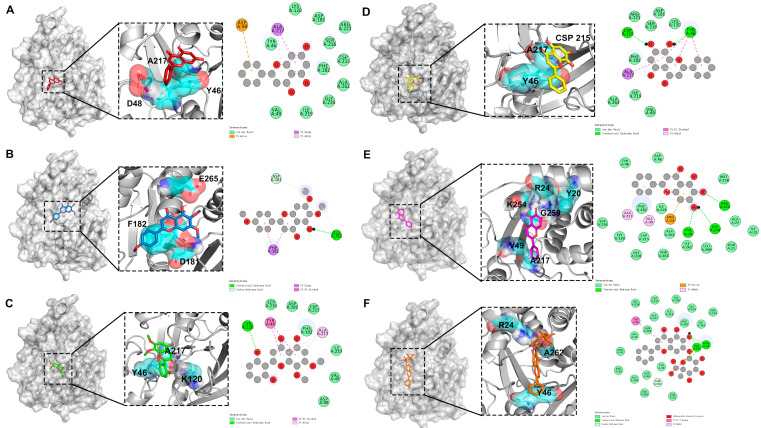
Molecular docking results of PTP1B and four active ingredients of Baikal skullcap. (**A**): The interaction between chrysin and PTP1B. (**B**): The interaction between salvigenin and PTP1B. (**C**): The interaction between 5,2′,6′-trihydroxy-7,8-dimethoxyflavone and PTP1B. (**D**): The interaction between norwogonin and PTP1B. (**E**): The interaction between baicalein and PTP1B. (**F**): The interaction between baicalin and PTP1B.

The molecular docking results identified key residues involved in the binding of various compounds to PTP1B, shown in Figure 5. For chrysin, residues A217, D48, and Y46 were pivotal for interaction. Trihydroxy-dimethoxyflavone primarily engaged with residues A217, Y46, and K120. The critical residues for the binding of salvigenin were E265, F182, and D181. Norwogonin interacted via CSP215, A217, and Y46, while baicalein formed key interactions with R24, K254, Y20, G259, V49, and A217. For baicalin, R24, A262, and Y46 were essential. Pi–Pi and Pi–alkyl interactions played a significant role in the binding of all four ligands, with tyrosine residues contributing substantially to the binding affinity. In molecular docking studies, the binding energy of baicalein was −7.7 kcal/mol, lower than −6.5 kcal/mol of baicalin, and lower than the other four active small molecules. Therefore, the results suggested a significant binding affinity of these compounds to the PTP1B enzyme, and the binding of baicalein to PTP1B was more stable, suggesting a superior inhibitory effect on PTP1B.

### 2.7. In Vitro Cellular Anti-Inflammatory Assays

We have chosen two molecules, baicalein and baicalin, for validation of their anti-inflammatory effects, which was motivated by several factors: beyond the proof through network pharmacology that these molecules have anti-inflammatory potential, the two molecules showed a higher affinity for PTP1B compared to the other four, enabling them to combat T2DM through various functions. Additionally, being the most abundant in Baikal skullcap, we gave priority to their experimental validation. The other two molecules used in the experiments are anti-inflammatory molecules from licorice, used as positive controls. 

**Figure 6:**
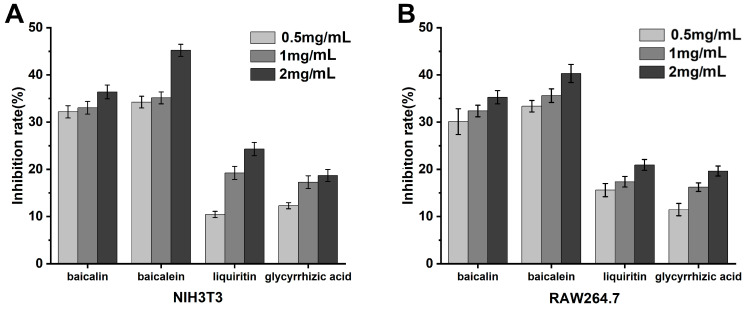
Detection of anti-inflammatory effect of T2DM traditional Chinese medicine extracts at the cellular level. (**A**): NIH3T3 cells. (**B**): RAW264.7 cells.

NIH3T3 Cell Assay: As indicated in Figure 6, in vitro cell experiments utilizing NIH3T3 cells to measure PGE2 concentrations revealed the anti-inflammatory efficacy of the herbal monomers. At concentrations above 1.0 mg/mL, the anti-inflammatory effects in vitro were ranked as follows: baicalein > baicalin > glycyrrhizin > glycyrrhetinic acid. Among all the active constituents, baicalein demonstrated the most potent anti-inflammatory effect, surpassing the anti-inflammatory activity of the bioactive components in licorice at all concentrations, suggesting that it has better inhibitory effects at all concentrations.

RAW264.7 Cell Assay: As demonstrated in Figure 6, in vitro experiments with RAW264.7 cells assessing NO concentrations indicated that the anti-inflammatory efficacy of the herbal monomers at concentrations greater than 1.0 mg/mL was in the order of baicalein > baicalin > glycyrrhizin > glycyrrhetinic acid. These data align with the anti-inflammatory outcomes of the NIH3T3 cell assay, with baicalein again showing the strongest anti-inflammatory effect among the monomers tested.

## 3. Discussion

This study focused on Baikal skullcap, employing LC–MS, in silico methods, and in vitro experimental techniques to explore the potential mechanisms through which its flavonoid active substances may treat type 2 diabetes mellitus (T2DM). Our results emphasized the significance of baicalein and baicalin as active components, highlighting their inhibitory effects on tyrosine phosphatase activity within the insulin signaling pathway, as well as their anti-inflammatory properties, thereby revealing potential mechanisms for their therapeutic application in T2DM.

Compared with other computational studies that explore the therapeutic mechanisms of herbs [23,24], our work integrated LC–MS results with database information, utilized various bioinformatics tools, and validated predicted interactions through molecular docking experiments. This approach provided a more comprehensive analysis, leading to a detailed understanding of the pharmacological mechanisms of Baikal skullcap’s effects on T2DM. Additionally, the predicted bioactivities were corroborated in biological systems by measuring the anti-inflammatory effects of baicalein and baicalin on NIH3T3 and RAW264.7 cell lines, providing experimental evidence for the predictions of network pharmacology. These efforts helped to ensure the biological significance of our findings and increased the reliability and accuracy of the research, contributing to a deeper understanding of the drug components’ mechanisms of action and improving the study’s reproducibility. Our research demonstrated the effective combination of dry and wet laboratory techniques, with each set of findings substantiating the other.

Despite these strengths, it is crucial to consider the limitations when interpreting the findings. Our research mainly focused on the flavonoid molecules represented by baicalein and baicalin, while the therapeutic action of Baikal skullcap might be the result of a synergistic effect of multiple components, and even trace molecules may be important. Furthermore, considering that T2DM is a complex disease, the impacts of drug molecules on various molecular targets and signaling pathways involved in its pathophysiology are also crucial, and this may represent a potential area for future research.

Moreover, potential side effects and safety issues associated with the use of baicalein and baicalin warrant consideration. Although flavonoids are typically associated with low toxicity and are generally considered safe, it is necessary to further evaluate their safety profile, particularly concerning long-term use and combination with other drugs or supplements. Conducting rigorous preclinical and clinical trials to assess the safety, efficacy, and optimal dosing strategies for baicalein and baicalin in diabetes treatment holds significant implications for future research. Such trials should encompass diverse populations and evaluate potential interactions with commonly used T2DM drugs such as glyburide and metformin.

In the future, the development of new analytical methods and experimental models will be required to capture the complex interactions accurately between the various components and targets of herbal medicines. This will help to clarify the synergistic effects of these substances, improve our understanding of their pharmacological mechanisms, and promote the development of more effective and personalized treatments for diabetes.

In summary, the present study has provided valuable insights into the active components of herbal medicine, especially regarding the potential pharmacological mechanisms by which Baikal skullcap can treat T2DM. By addressing the limitations and challenges identified, future research can further advance our comprehension regarding the complex interactions between traditional medicine and modern scientific methods, ultimately contributing to the development of safe, effective, and personalized treatments for diabetes and related diseases.

## 4. Materials and Methods

### 4.1. LC–MS Experiments

We used licorice as a positive control. Baikal skullcap and licorice were supplied by our laboratory; 95% ethanol and acetone (analytical grade) were obtained from Beijing Chemical Works(Beijing, China). Fifty grams of the powdered Baikal skullcap and licorice were each measured and subjected to a freeze-drying process to obtain the extracts. Mass spectrometry analysis identified the primary components in these two herbs as baicalin, baicalein, glycyrrhizin, and glycyrrhetinic acid. The monocomponent main constituents of baicalin, baicalein, glycyrrhizin, and glycyrrhetinic acid were purchased from Xi’an Kaili Biotechnology Co., Ltd. (Xi’an, China), with a purity of over 90%. Liquid chromatography–mass spectrometry (LC–MS) was employed to identify and verify the composition and purity of the purchased pharmaceuticals. The equipment used included a Finnigan LTQ XL ion trap mass spectrometer with an ESI electrospray ion source, and an Accela U-HPLC high-performance liquid chromatography system, which features an Accela temperature-controlled autosampler, column oven, Accela 1250 ultra-high pressure liquid pump, and Accela photodiode array detector, all of which are products purchased from Thermo Fisher Scientific, Waltham, MA, USA.

### 4.2. In Silico Experiments

#### 4.2.1. Predictive Analysis of T2DM Potential Targets and Baikal Skullcap Component Interactions

In our research, we embarked on identifying target genes linked to type 2 diabetes mellitus (T2DM) by consulting four renowned databases: DisGeNET [25], GeneCards [26], PharmGKB [27], and UniProt [28]. From DisGeNET, employing a rigorous criterion with a threshold score above 0.1, we discerned 591 target genes associated with T2DM. The GeneCards repository revealed 1567 targets, requiring a minimal score of 5 for inclusion, whereas PharmGKB presented 41 specific target genes. Through meticulous comparison and deduplication among these sources, we distilled the list to 1410 unique target genes for subsequent investigations. To ascertain potential interactions between the active components in Baikal skullcap and these earmarked T2DM genes, we utilized tools and databases such as SEA [29], Super-Pred [30], and SwissTargetPrediction [31]. This analytical process was aimed at making thorough predictions on the cross-targeting potential of various active components like baicalein, baicalin, chrysin, among others. Our efforts culminated in identifying 253 pertinent cross-target genes for further scrutiny.

#### 4.2.2. Precise Construction of the Protein–Protein Interaction Network

Employing the STRING database [32], a distinguished bioinformatics resource, we developed an extensive protein–protein interaction (PPI) network. This endeavor aimed to explore the intricate relationships among crucial target proteins previously identified and filtered. For visualization, we leveraged the most recent iteration of Cytoscape3.9.0 software [33]. Additionally, we undertook a detailed examination of the network’s topological characteristics utilizing Cytoscape’s sophisticated analytical capabilities. This meticulous analysis led to the identification of ten hub genes, which play critical roles in the underlying mechanisms of the disease.

#### 4.2.3. Comprehensive Enrichment Analysis of GO and KEGG Pathways

In our research, we extensively utilized an array of R packages, including BiocManager and clusterProfiler, to conduct enrichment analyses focused on Gene Ontology (GO) [34,35] biological processes and Kyoto Encyclopedia of Genes and Genomes (KEGG) [36] pathways. Our goal was to uncover the roles and interactions of target genes within biological frameworks. We applied strict criteria for statistical significance, mandating both *p* and q values to be below 0.01. These procedures allowed us to extract comprehensive GO data from the tissue, which were subsequently presented through bar and bubble charts. This approach facilitated clearer visualization, enhancing the comprehension and interpretation of our findings.

#### 4.2.4. Quantum Chemical Calculations and Molecular Docking of Active Components in Baikal Skullcap

In the aspect of quantum chemical calculations, we chose Gaussian 09 software along with the GaussView 5.0 tool for precise quantitative analysis. We applied the B3LYP method from Density Functional Theory (DFT) [37], along with the 6–31G basis set, to calculate the electronic structure of six major active components in Baikal skullcap, such as the Highest Occupied Molecular Orbital (HOMO) and Lowest Unoccupied Molecular Orbital (LUMO). Multiwfn [38] was used to plot visualization graphs for quantitative calculations, and we created detailed visual charts for analysis and discussion. Subsequently, molecular docking was performed with Autodock Vina 1.2.0 [39]. The structures with the lowest energy were selected from the docking results. Based on the PPI analysis results, the six active components of Baikal skullcap also target the gene *PTPN1* encoding the protein PTP1B. From the RCSB Protein Data Bank [40], the crystal structure of PTP1B (4I8N) [41] was chosen as the receptor protein for further molecular docking. Before docking, ligands and water molecules were removed from the crystal structure of PTP1B to prepare the protein. Since there is no crystal structure of PTP1B with an active small molecule, the active site of PTP1B bound with a peptide was chosen to define the molecular docking site. Then, the six active components of Baikal skullcap were docked as ligands.

### 4.3. In Vitro Cellular Anti-Inflammatory Assays

We prioritized the experimental validation of baicalein and baicalin, the most abundant components in *Scutellaria baicalensis*, for their anti-inflammatory properties. Additionally, glycyrrhizin and glycyrrhetinic acid, the anti-inflammatory molecules from licorice, were used as positive controls. Initially, cell revival and passaging were conducted. Cells were swiftly retrieved from −80 °C storage and thawed in a 37 °C water bath. Upon complete melting, they were centrifuged at 1000 rpm for 5 min. The supernatant was discarded, and the pellet was resuspended in 1 mL of culture medium. Cells were then seeded into culture dishes, to which 9 mL of culture medium and 1 mL of serum were added to continue culturing. When cell confluence reached 80%, cells were digested with 2 mL of trypsin, followed by quenching with 1 mL of serum and 3 mL of culture medium, then centrifuged and equally distributed into new culture dishes. An MTT assay [42] was performed to assess cell proliferation activity, where cells were cultured with various herb extracts at a final concentration of 1 mg/mL. The NIH3T3 cells [43] (mouse embryonic fibroblasts) and RAW264.7 cells (mouse macrophages) used in the experiments were both purchased from Nanjing KeyGen Biotech Co., Ltd., Nanjing, China.

NIH3T3 Cell Assay

Cells were allocated into a 96-well plate (New England Biolabs, Ipswich, MA, USA) at a density of 10^5^ cells per well, and 200 μL of Dulbecco’s Modified Eagle Medium (DMEM) with 1% (*v*/*v*) penicillin/streptomycin as well as containing 10% fetal bovine serum (FBS) was added. Cells were incubated at 37 °C in a 5% CO_2_ atmosphere for 24 h. The medium was then replaced with DMEM containing 0.5% FBS and incubated for an additional 24 h. Cells were treated with 1 mmol/L aspirin (purchased from Shanghai Aladdin Company, Shanghai, China) for 30 min, washed with PBS, and then new culture medium was added for further cultivation. Control group received no medication, while test groups were administered various concentrations of herb small molecules (0.5, 1, and 2 mg/mL) and incubated at 37 °C in 5% CO_2_ for 30 min. Each group was then treated with a final concentration of 2 μmol/L arachidonic acid (purchased from Beyotime Institute of Biotechnology, Shanghai, China) and incubated at 30 °C in 5% CO_2_ for 15 min. Three sets of replicate experiments were established. Subsequent operations followed the ELISA kit (Beyotime Institute of Biotechnology, Shanghai, China) instructions, and prostaglandin E2 (PGE2) secretion was measured.

RAW264.7 Cell Assay

RAW264.7 cells were cultured in RPMI 1640 (purchased from Hyclone, Canton, MA, USA) medium supplemented with 10% serum until sufficient growth was achieved for passaging and cryopreservation. Log-phase RAW264.7 cells were transferred into a 24-well plate at 30,000 cells per well and cultured in RPMI 1640 with 10% serum, with a total volume of 50 μL per well for 1 h. Different concentrations of herb small molecules (0.5, 1, and 2 mg/mL) were added according to experimental requirements and incubated for an additional 3 h. The control group treated with LPS purchased from Sigma-Aldrich, St. Louis, MO, USA, was administered a final concentration of 1 μg/mL LPS for 18 h. Then, three sets of replicate experiments were established. NO assay kits (Beyotime Institute of Biotechnology, Shanghai, China) were taken from a 4 °C refrigerator and allowed to reach room temperature for 15–30 min before following the kit instructions for subsequent operations. Nitric oxide (NO) secretion was then measured.

## 5. Conclusions

This investigation into Baikal skullcap has unearthed a suite of six flavonoids, with baicalein and baicalin presenting promising therapeutic potential for T2DM by modulating PTP1B activity and exerting potent anti-inflammatory effects. Network pharmacology revealed their potential anti-inflammatory and PTP1B modulatory effects. Molecular docking confirmed baicalein and baicalin as promising PTP1B binding components. In cell assays and paw swelling experiments, baicalein and baicalin exhibited superior anti-inflammatory properties over glycyrrhizin and glycyrrhetinic acid. Two compounds, baicalein and baicalin, show potential for pharmacological modification, with the remaining four meriting further investigation for similar prospects. Collectively, these findings bolster the therapeutic repertoire against T2DM and suggest a compelling case for the integration of phytochemicals from herbs into modern pharmacological applications.

## Figures and Tables

**Table 1:** The six active ingredients of Baikal skullcap.

Molecule Name	MW	OB (%)	DL
chrysin	254.25	22.61	0.18
baicalein	270.25	33.52	0.21
Salvigenin	328.34	49.07	0.33
5,2′,6′-Trihydroxy-7,8-dimethoxyflavone	330.31	45.05	0.33
Norwogonin	270.25	39.4	0.21
Baicalin	460.42	29.53	0.77

## Data Availability

Data is contained within the article and Supplementary Materials.

## Supplementary Materials

The following supporting information can be downloaded at https://www.mdpi.com/article/10.3390/ijms25073654/s1.
