# Supplementary material for: Unveiling Anti-Diabetic Potential of Baicalin and Baicalein from Baikal Skullcap: LC–MS, In Silico, and In Vitro Studies
Source: Int J Mol Sci. 2024 Mar 25;25(7):3654. doi: 10.3390/ijms25073654 (PMC11011639; doi:10.3390/ijms25073654)
Supplement: Supplementary file 1 [file ijms-25-03654-s001.zip › ijms-2921642-supplementary.pdf]

Unveiling Anti-Diabetic Potential of Baicalin and Baicalein from Baikal Skullcap: LC-MS, in Silico and in Vitro studies

Supporting Information

Wencheng Zhao[†], Huizi Cui[†], Kaifeng Liu, Xiaotang Yang, Shu Xing* and Wannan Li*

Edmond H. Fischer Signal Transduction Laboratory, Key Laboratory for Molecular Enzymology and Engineering of Ministry of Education, School of Life Sciences, Jilin University, Changchun 130012, China

* Correspondence: xingshu@jlu.edu.cn (S.X.); liwannan@jlu.edu.cn (W.L.)

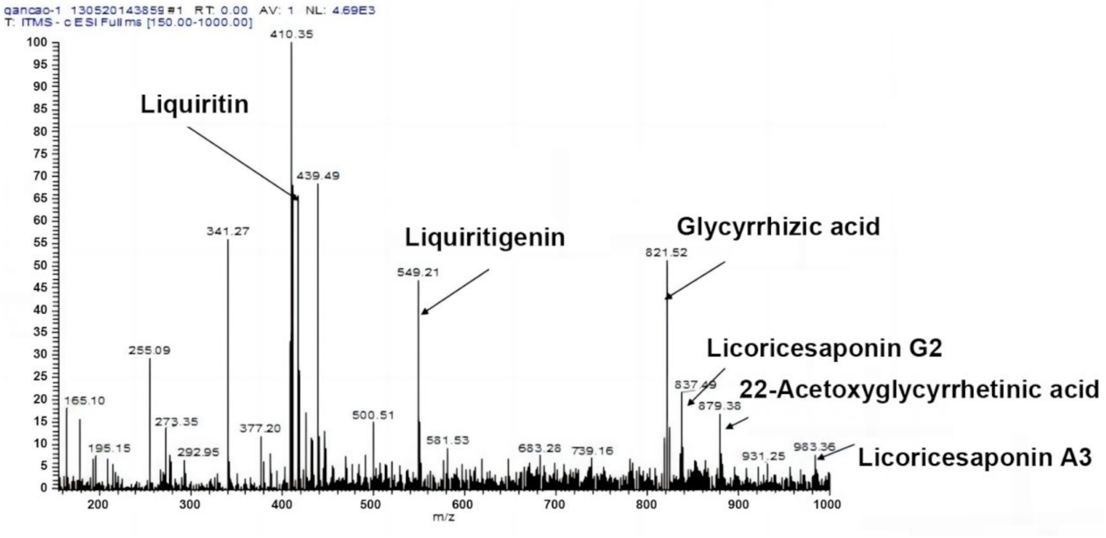

Figure S1. Mass spectrometry detection of licorice

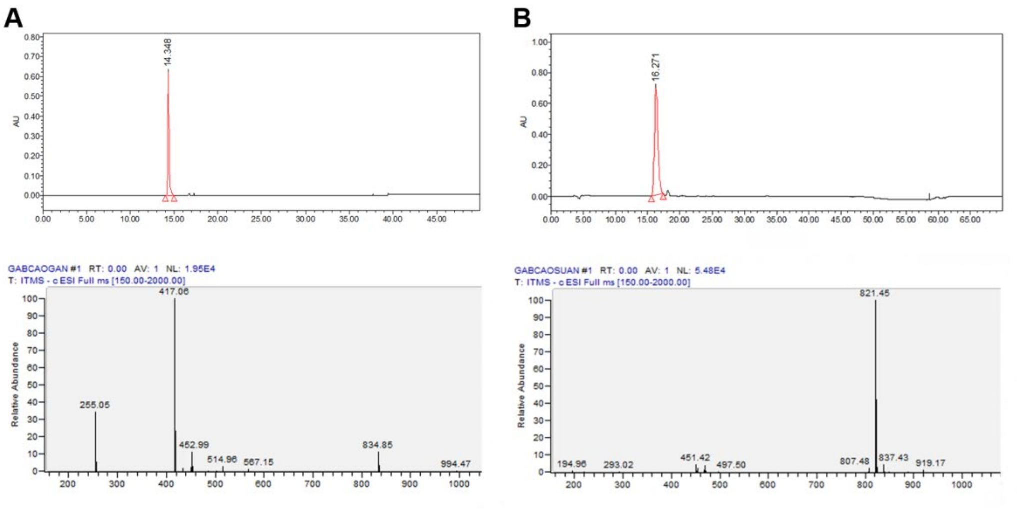

Figure S2. Liquid phase and mass spectrometry detection of liquiritin and glycyrrhizic acid purity. A: The liquid phase and mass spectrum of liquiritin. B: The liquid phase and mass spectrum of glycyrrhizic acid.

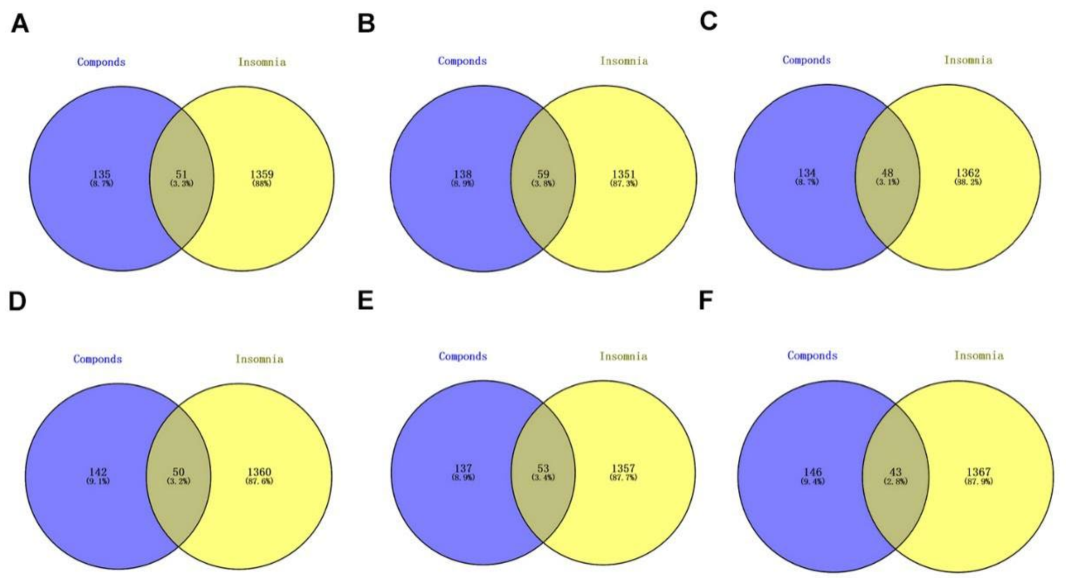

Figure S3. Venn diagram reveals shared intersection genes between Scutellaria baicalensis and T2DM. A: The number of common genes shared between chrysin and T2DM is 51. B: The number of common genes shared between Salvigenin and T2DM is 59, C: The number of common genes shared between 5,2',6'-Trihydroxy-7,8-dimethoxyflavone and T2DM is 51. D: The number of common genes shared between Norwogonin and T2DM is 51. E: The number of common genes shared between Baicalein and T2DM is 51. F: The number of common genes shared between Baicalin and T2DM is 51.

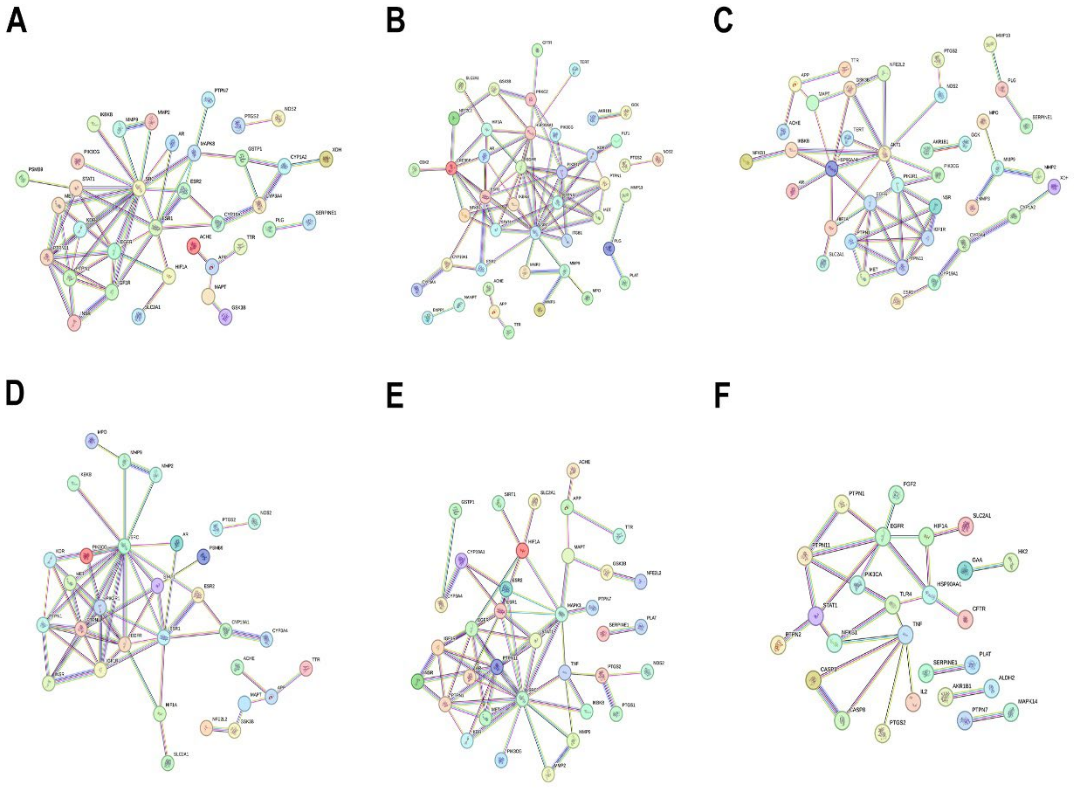

Figure S4. PPI network analysis using the STRING database demonstrates the interaction of intersection genes found in Scutellaria baicalensis and T2DM.

A: PPI network of Chrysin, B: PPI network of Salvigenin. C: PPI network of 5,2',6'-Trihydroxy-7,8-dimethoxyflavone. D: PPI network of Norwogonin. E: PPI network of Baicalein. F: PPI network of Baicalin.

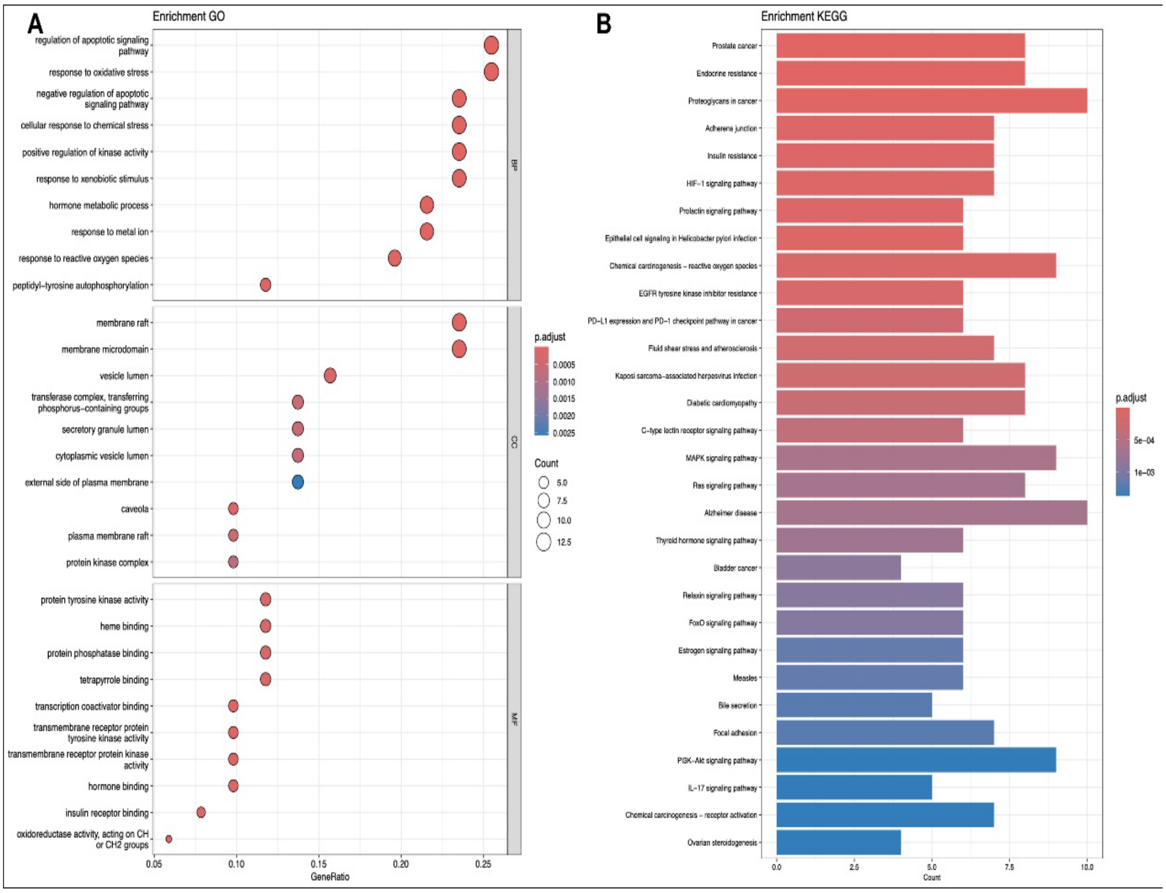

Figure S5. Results of GO and KEGG pathway enrichment analysis based on genes shared by Chrysin and T2DM. Visualization of GO analysis related to Chrysin against T2DM, including biological processes, molecular functions, and cellular components. KEGG pathway enrichment analysis results of Chrysin and T2DM intersection genes. The T2DM justed p-value is represented by the bar color; the size of the bubble represents the number of genes.

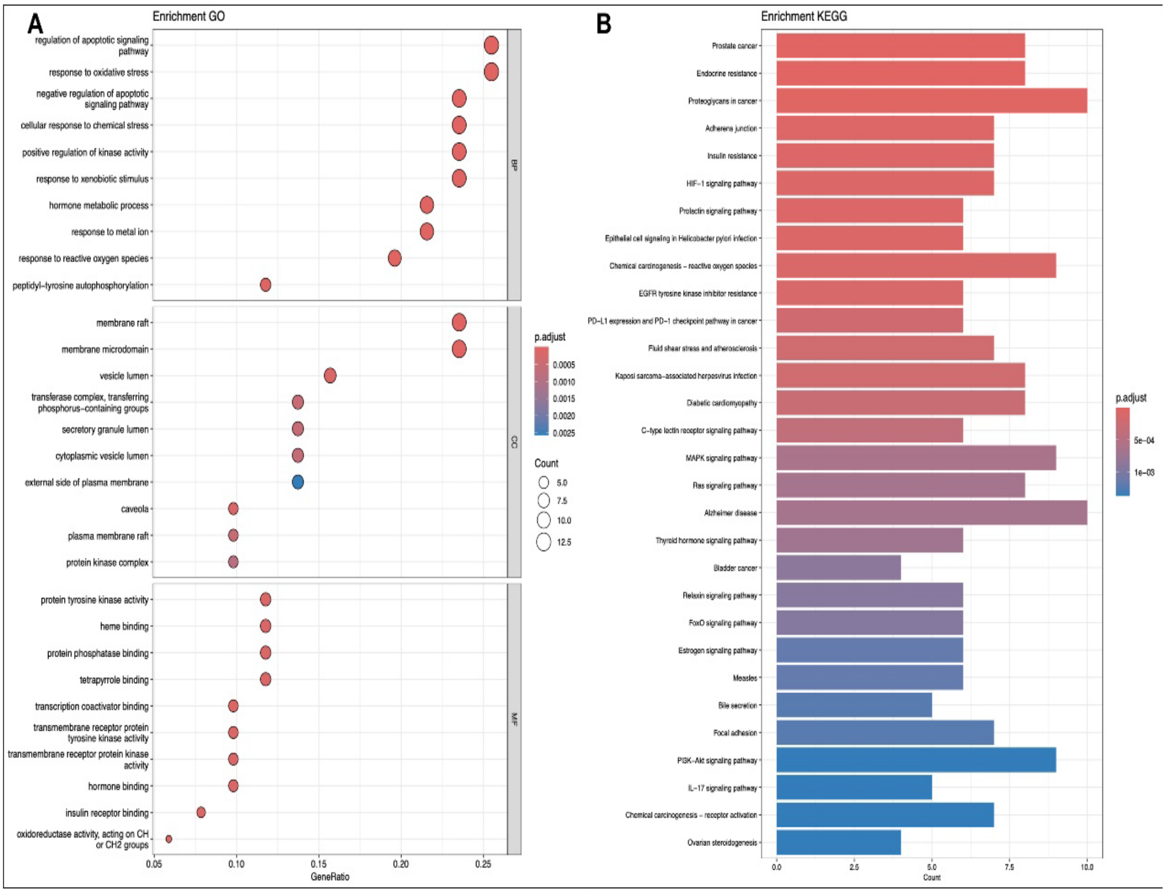

Figure S6. Results of GO and KEGG pathway enrichment analysis based on genes shared by Salvigenin and T2DM. Visualization of GO analysis related to Salvigenin against T2DM, including biological processes, molecular functions, and cellular components. KEGG pathway enrichment analysis results of Salvigenin and T2DM intersection genes. The T2DM justed p-value is represented by the bar color; the size of the bubble represents the number of genes.

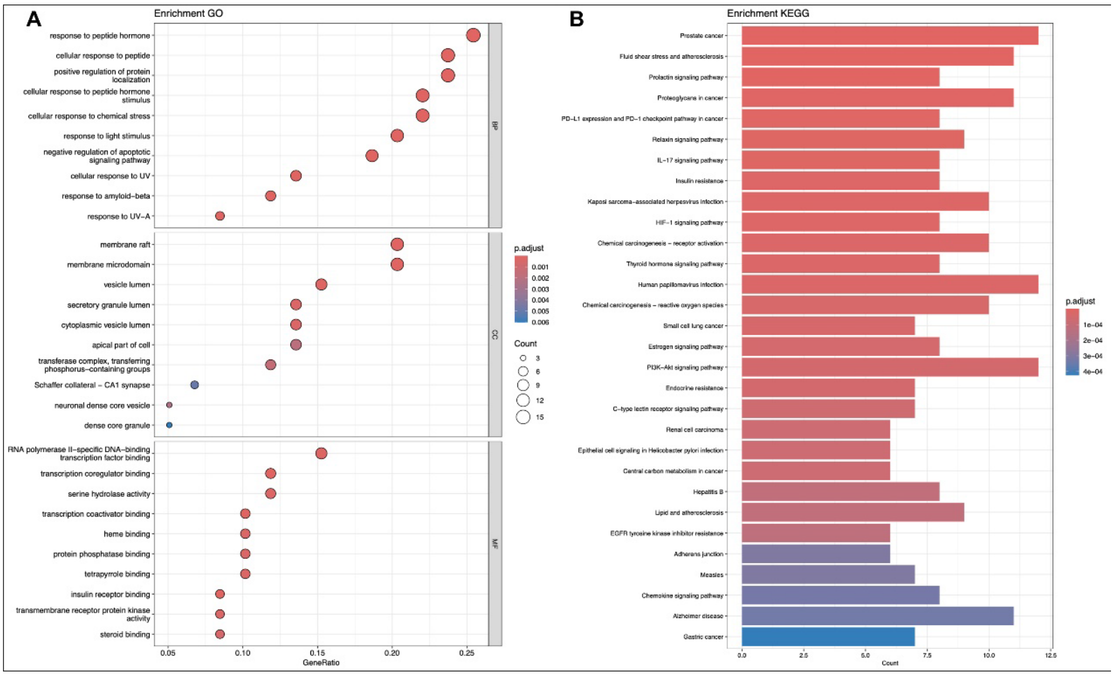

Figure S7. Results of GO and KEGG pathway enrichment analysis based on genes shared by 5,2',6'-Trihydroxy-7,8-dimethoxyflavone and T2DM. Visualization of GO analysis related to 5,2',6'-Trihydroxy-7,8-dimethoxyflavone against T2DM, including biological processes, molecular functions, and cellular components. KEGG pathway enrichment analysis results of 5,2',6'-Trihydroxy-7,8-dimethoxyflavone and T2DM intersection genes. The T2DM justed p-value is represented by the bar color; the size of the bubble represents the number of genes.

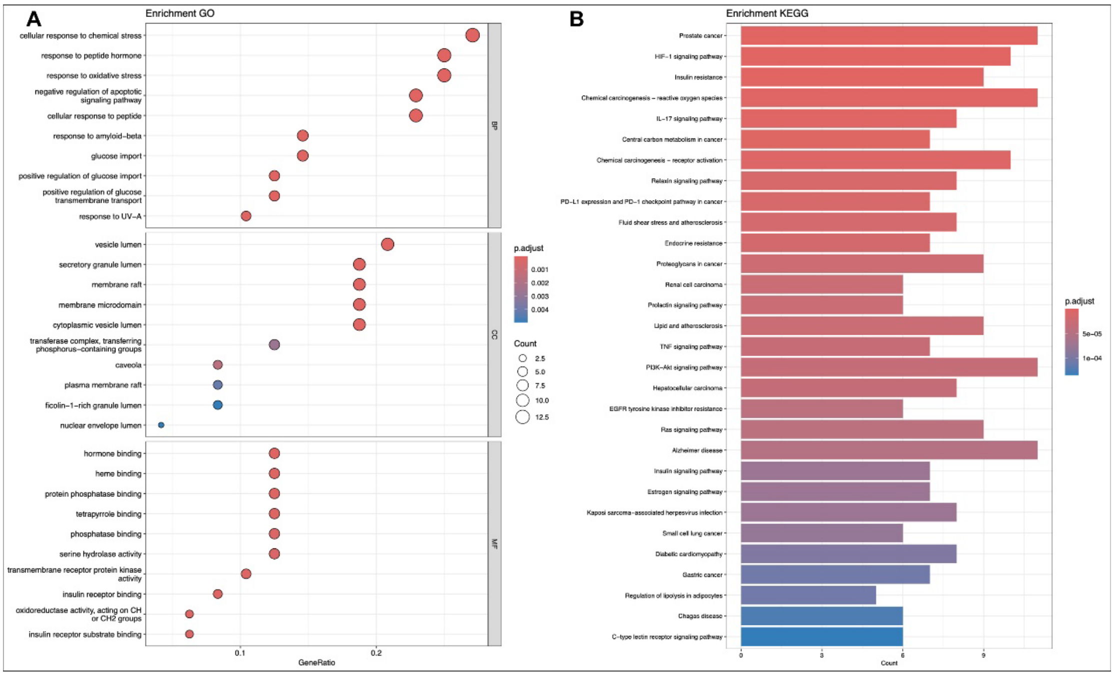

Figure S8. Results of GO and KEGG pathway enrichment analysis based on genes shared by Norwogonin and T2DM. Visualization of GO analysis related to Norwogonin against T2DM, including biological processes, molecular functions, and cellular components. KEGG pathway enrichment analysis results of Norwogonin and T2DM intersection genes. The T2DM justed p-value is represented by the bar color; the size of the bubble represents the number of genes.

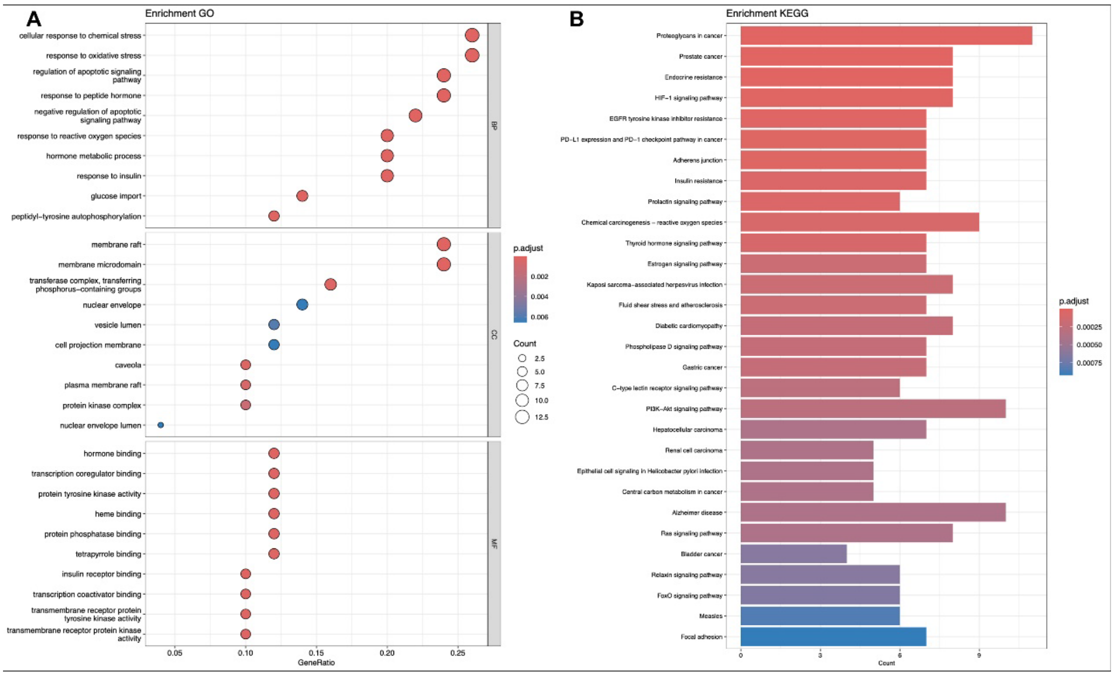

Figure S9. Results of GO and KEGG pathway enrichment analysis based on genes shared by Baicalein and T2DM. Visualization of GO analysis related to Baicalein against T2DM, including biological processes, molecular functions, and cellular components. KEGG pathway enrichment analysis results of Baicalein and T2DM intersection genes. The T2DMjusted p-value is represented by the bar color; the size of the bubble represents the number of genes.

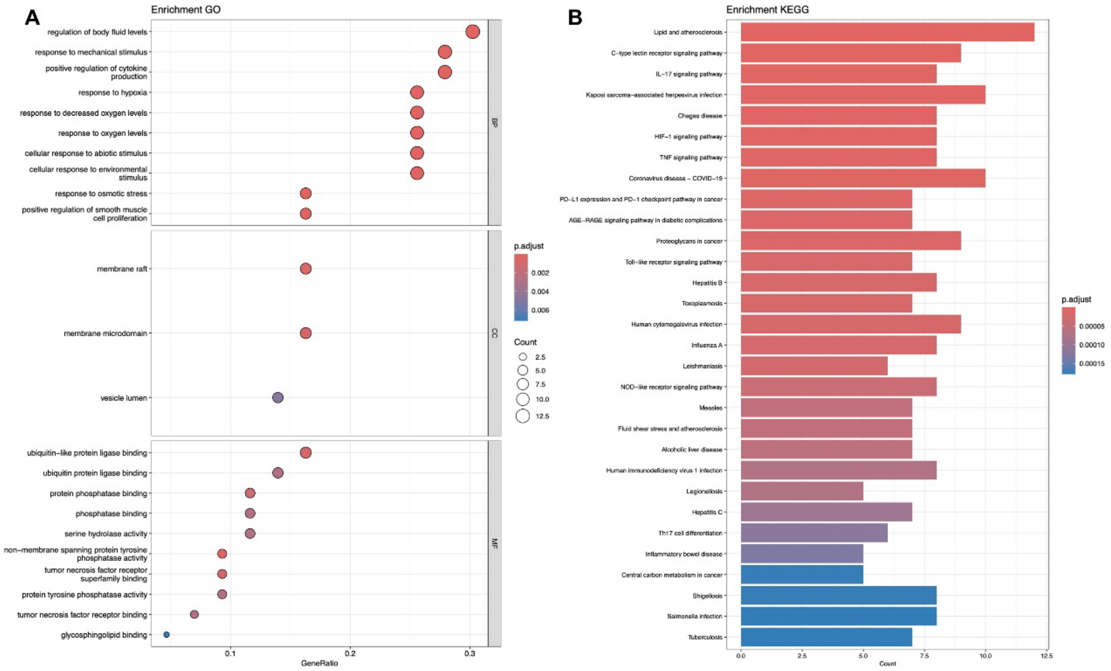

Figure S10. Results of GO and KEGG pathway enrichment analysis based on genes shared by Baicalin and T2DM. Visualization of GO analysis related to Baicalin against T2DM, including biological processes, molecular functions, and cellular components. KEGG pathway enrichment analysis results of Baicalin and T2DM intersection genes. The T2DM justed p-value is represented by the bar color; the size of the bubble represents the number of genes.
